# Research on Trajectory Tracking Control of Inspection UAV Based on Real-Time Sensor Data

**DOI:** 10.3390/s22103648

**Published:** 2022-05-11

**Authors:** Mingbo Yang, Ziyang Zhou, Xiangming You

**Affiliations:** Department of Mechanical and Electronic Engineering, School of Mechanical and Material Engineering, North China University of Technology, Beijing 100144, China; zhouziyangzzy11@126.com (Z.Z.); youxiangming@mail.ncut.edu.cn (X.Y.)

**Keywords:** inspection UAVs, trajectory tracking control, stability, robustness, sensor data

## Abstract

In power inspection, uncertainties, such as wind gusts in the working environment, affect the trajectory of the inspection UAV (unmanned aerial vehicle), and a sliding mode adaptive robust control algorithm is proposed in this paper to solve this problem. For the nonlinear and under-driven characteristics of the inspection UAV system, a double closed-loop control system which includes a position loop and attitude loop is designed. Lyapunov stability analysis is used to determine whether the designed system could finally achieve asymptotic stability. Sliding-mode PID control and a backstepping control algorithm are applied to analyze the superiority of the control algorithm proposed in this paper. A PX4 based experimental platform system is built and experimental tests were carried out under outdoor environment. The effectiveness and superiority of the control algorithm are proposed in this paper. The experimental results show that the sliding mode PID control can achieve good accuracy with smaller computing costs. For nonlinear interference, the sliding mode adaptive robust control strategy can achieve higher trajectory tracking accuracy.

## 1. Introduction

**Figure 1:**
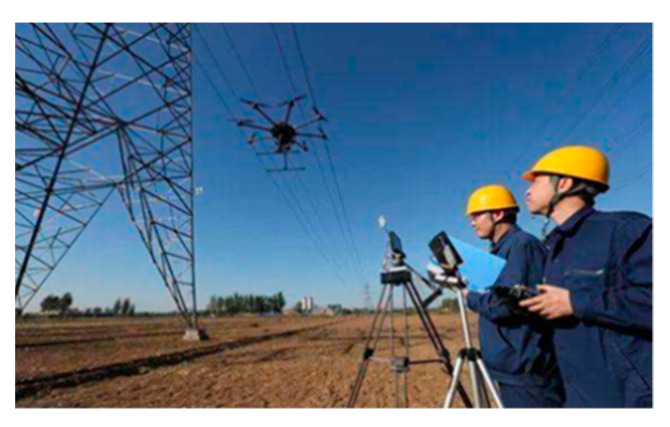
Inspection drone operations.

Since the second industrial revolution, electricity has played an indispensable role in human life. China has a vast territory, and overhead transmission lines cover most of it for power transmission. However, most of these transmission networks are built outdoors and are vulnerable to bird nesting, thunder, and lightning. Therefore, it is necessary to conduct power inspections on the transmission line network. At present, China mainly uses electric inspection drones for this project. Compared with manual inspections, inspection drones can improve inspection efficiency and enhance safety [1,2,3]. As shown in Figure 1, electric power workers are conducting electric inspection work by use inspection drone.

**Figure 2:**
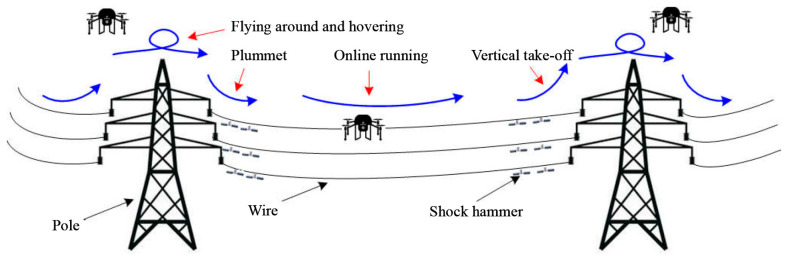
Schematic diagram of patrol tracking track.

In the power inspection project, the inspection drone works according to the inspection trajectory established by the staff, as shown in Figure 2. However, due to the interference of factors such as wind gusts at work, the inspection UAV has deviations in the process of tracking the inspection trajectory. Therefore, it is very necessary to design a controller that can make the patrol unmanned and track the patrol track stably.

At present, researchers have proposed the following control algorithms for the trajectory tracking control of the inspection drone: PID control, adaptive control, backstepping control, active disturbance rejection control, sliding mode control, etc. [4,5,6,7]. The PID control structure is simple, but the process of adjusting the parameters is relatively cumbersome and the robustness is poor.

Literature [8,9] uses PID control algorithm to design the controller. The error is calculated by the data obtained by the position and attitude sensor and theoretical data, the PID parameters are adjusted to make the error approach zero, and the flight trajectory of the UAV can be tracked. However, there are two main disadvantages. On the one hand, the process of adjusting parameters is complicated, while on the other hand, the robustness is poor, and it is difficult to achieve high-precision trajectory tracking. Literature [10] proposed the use of back-stepping control to design the trajectory tracking controller, establishing the position and attitude error dynamic model of the UAV, using the backstepping method to design the stabilization control items, and using Lyapunov theory to prove the closed-loop system stability, the final trajectory tracking experiment proved the effectiveness of the control algorithm. Although the system is stable, after disturbance is added, the tracking effect becomes worse, and the anti-interference ability of the system is not strong. In [11], the extended state observer of the switching function is used to estimate the disturbance in trajectory tracking, and the calculated estimated value is combined with sliding mode control to finally achieve the trajectory tracking effect. However, chattering occurs in sliding mode control, and the tracking accuracy is affected. Literature [12] uses dual-loop control to achieve high-precision trajectory tracking. The inner loop adopts sliding mode control, while the outer loop adopts linear auto-disturbance rejection control to suppress external interference. Experiments have proven that this method has fast response speed, strong robustness, and can meet the needs of trajectory tracking control of inspection drones. In the literature [13,14,15], fuzzy control is used to design the controller, and the fuzzy system is used to gradually approximate the uncertain items and external interference in the attitude model, while the controller is designed in combination with back-stepping control, which improves the accuracy of trajectory tracking. In recent years, neural network control has begun to increase in popularity, and it has also been favored by scholars. In the literature [16,17,18], facing the uncertainty of the inspection UAV model, the neural network control is used to approximate, and then the external disturbance is suppressed by the observer. Finally, combined with the backstep control, the trajectory tracking effect of the attitude and position of the inspection drone is well achieved.

This paper addresses the problem whereby the inspection UAV is disturbed by unknown factors, such as wind gusts in the trajectory tracking process, resulting in the inability to track the inspection trajectory with high accuracy. In this paper, a sliding mode adaptive robust control algorithm is used to design the controller. Compared with other control algorithms, the adaptive algorithm part can better suppress disturbances, while the sliding mode control part can enhance the stability of the system and compensate for the poor robustness of other control algorithms. A control group experiment was also conducted using the sliding mode PID control algorithm and the backstepping control algorithm, and the simulation results and experimental results proved that, in terms of control structure, the method in this paper is more concise, efficient, and has certain advantages compared with other control methods that require the design of an observer or compensator and the repeated adjustment of control parameters.

## 2. Inspection Drone Dynamic Model

### 2.1. Establishment of Coordinate System

**Figure 3:**
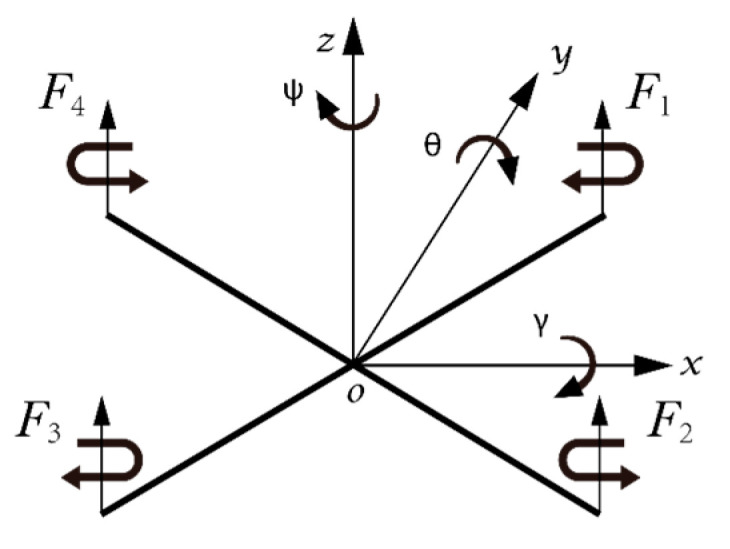
Establishment of four rotor aircraft coordinate system.

In order to better study the motion state of inspection drone dynamic model, it is necessary to select the appropriate coordinate system. In this paper, the body axis system and inertial coordinate system are introduced to describe the rotation, position, and attitude of the aircraft. The body axis system is *A*($A_{x}$, $A_{y}$, $A_{z}$). The inertial coordinate system is *B*($B_{x},\;\;B_{y}$, $B_{z}$). As shown in Figure 3, *γ*, *θ,* and ψ are roll angle, pitch angle, and yaw angle, respectively. Through the rotation matrix of three angles, the transformation matrix from body axis system *A* to inertial coordinate system *B* can be deduced $R_{AB}$ [19].

(1) $$R_{AB}\;=R_{\gamma }R_{\theta }R_{\psi }=\left[\begin{array}{ccc} C\theta C\psi & S\theta S\gamma C\psi -S\psi C\gamma & C\psi C\gamma S\theta +S\gamma S\psi \\ C\theta S\psi & S\theta S\gamma S\psi +C\psi C\gamma & S\theta S\psi C\gamma -S\gamma C\psi \\ -S\theta & C\theta S\gamma & C\theta C\gamma \end{array}\right]$$

### 2.2. Establishment of Coordinate System

In order to facilitate the establishment of the dynamic model, the following assumptions are proposed:

1. The four-rotor aircraft is regarded as a rigid body, and its body structure is completely uniform and symmetrical.
2. The origin *O* of the airframe coordinate system coincides with the origin *O* of the inertial coordinate system to ensure that the inertial matrix is a diagonal matrix in the rigid body coordinate system [20].

Because the main motion forms of quadrotor aircraft are divided into two types, one is translation motion relative to inertial frame *B*, while the other is rotation relative to body axis system *A*. Translation has 3 degrees of freedom, rotation has 3 degrees of freedom, a total of 6 degrees of freedom. Therefore, according to the total degrees of freedom, the generalized coordinates can be determined as ***q*** = ${\left(\xi ,\eta \right)}^{T}$, where ξ = [*x*,*y*,*z*]^T^ represents location information; η  = [ γ, θ, ψ]^T^ indicates attitude information. The mass of the aircraft is *m*, the acceleration of gravity is *g*, and the distance from the end of the rotor to the center of gravity of the aircraft is *l*. The inertia matrix of quadrotor aircraft in the body axis system is as follows:

$$I\;=\;\left[\begin{array}{ccc} I_{x} & 0 & 0\\ 0 & I_{y} & 0\\ 0 & 0 & I_{z} \end{array}\right]$$

where $I_{x},\;I_{y},\;I_{z}$ represent the moment of inertia of the *x* axis, *y* axis, and *z* axis in a coordinate system, which can be obtained by testing [21].

The expression equation of position part of quadrotor aircraft is as follows:

(2) $$m\ddot{\xi }=\;u_{1}R_{AB}e_{3}-\mathrm{mg}e_{3}+d_{s}$$

where $e_{3}\;$=$\;{\left[\begin{array}{ccc} 0 & 0 & 1 \end{array}\right]}^{T},\;d_{s}$ is the disturbing force of the moving part of the position, $u_{1}$ is the lift power of a four rotor aircraft, $R_{AB}$ is a transformation matrix, and $d_{s}\;$= ${\left[\begin{array}{ccc} d_{x} & d_{y} & d_{z} \end{array}\right]}^{T}$ is the interference force in the translational motion.

The equations for the rotational part of the quadrotor aircraft are as follows:

(3) $$J\;\ddot{\eta }\;+\;C\dot{\eta }=\;\tau +d_{M}$$

where ***τ*** is the moment of rolling, pitching and yawing, ***τ*** = ${\left[\begin{array}{ccc} \tau_{\gamma } & \tau_{\theta } & \tau_{\psi } \end{array}\right]}^{T}$. $d_{M}$ = ${\left[\begin{array}{ccc} d_{\gamma } & d_{\theta } & d_{\psi } \end{array}\right]}^{T}$; is the disturbing torque in the rotational motion; ***J*** is the representation of the moment of inertia I of the rigid body in *B* coordinate system.

(4) $$J=\left[\begin{array}{ccc} I_{x} & 0 & -I_{x}S\theta \\ 0 & I_{y}{C\gamma }^{2}+I_{z}{S\gamma }^{2} & \left(I_{y}-I_{z}\right)S\gamma C\gamma C\theta \\ -I_{x}S\theta & \left(I_{y}-I_{z}\right)S\gamma C\gamma C\theta & I_{x}S\theta^{2}+I_{y}{S\gamma }^{2}C\theta^{2}+I_{z}{C\gamma }^{2}C\theta^{2} \end{array}\right]$$

The coefficient ***C*** is the Coriolis force and centrifugal term [22], which is expressed in the following form [23].

(5) $$C=\left[\begin{array}{ccc} C_{11} & C_{12} & C_{13}\\ C_{21} & C_{22} & C_{23}\\ C_{31} & C_{32} & C_{33} \end{array}\right]$$

$$\begin{array}{l} C_{11}=0\\ C_{12}=-I_{x}\dot{\psi }C\theta +\left(I_{y}-I_{z}\right)(\;\dot{\theta }S\gamma C\gamma +\psi {S\gamma }^{2}C\theta -\dot{\psi }{C\gamma }^{2}C\theta )\\ C_{13}=\left(I_{z}-I_{y}\right)\;\dot{\psi }S\gamma C\gamma C\theta^{2}\\ C_{21}=-C_{12}\\ C_{22}=\left(I_{z}-I_{y}\right)\;\dot{\gamma }\;S\gamma C\gamma \\ C_{23}=-I_{x}\dot{\psi }C\theta S\theta +I_{y}\dot{\psi }C\theta S\theta {S\gamma }^{2}+I_{z}\dot{\psi }C\theta S\theta {C\gamma }^{2}\\ C_{31}=\left(I_{y}-I_{z}\right)\dot{\psi }S\gamma C\gamma -I_{x}\dot{\theta }C\theta \\ C_{32}=\left(I_{z}-I_{y}\right)(\dot{\theta }S\gamma C\gamma S\theta +{\dot{\gamma }\;S\gamma }^{2}C\theta )+\left(I_{y}-I_{z}\right){\dot{\gamma }\;C\gamma }^{2}C\theta +I_{x}\dot{\psi }C\theta S\theta -I_{y}\dot{\psi }{S\gamma }^{2}C\theta S\theta -I_{z}\dot{\psi }C\gamma^{2}C\theta S\theta \\ C_{33}=\left(I_{y}-I_{z}\right)\dot{\gamma }\;S\gamma C\gamma C\theta^{2}-I_{y}\dot{\theta }{S\gamma }^{2}C\theta S\theta -I_{z}\dot{\theta }{C\gamma }^{2}C\theta S\theta +I_{x}\dot{\theta }C\theta S\theta \end{array}$$

## 3. Control System Design

### 3.1. Controller Design Structure

**Figure 4:**
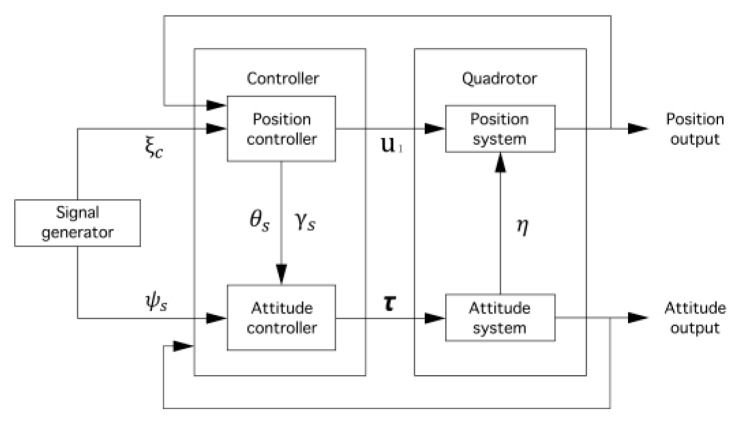
Establishment of four rotor aircraft coordinate system.

In this paper, a sliding mode adaptive robust trajectory tracking control strategy for quadrotor aircraft is proposed. The system is divided into two dynamic subsystems: position subsystem and attitude subsystem. The position subsystem is taken as the outer loop subsystem, and the attitude part is taken as the inner loop subsystem. The overall design scheme is shown in Figure 4.

### 3.2. Position Controller Design

The main design idea of the position controller is to compare the ideal position trajectory with the actual position trajectory, and finally make the error of the two approaches to zero.

Thus, the desired tracking displacement trajectory can be set as $\xi_{c}$, The displacement obtained by actual tracking is recorded as ξ. Set the error to $e_{s}$, where $\;e_{s}$= $\xi -\xi_{c}$. According to Formula (2), $\ddot{\xi }$ can be expressed separately. Then, find the two derivatives on both sides of the error expression as follows:

(6) $$\ddot{e_{s}}\;=\;\frac{u_{s}+\;d_{s}\;}{m}-\;ge_{3}-\ddot{\xi_{c}}$$

In the formula, $u_{s}\;$= $u_{1}R_{AB}e_{3}$ is the control input that needs to be designed,$\;u_{s}$ is understood as a virtual control law, as an intermediate quantity of coordinate transfomation.

According to the sliding mode adaptive robust control method, the sliding mode function is designed first. The sliding mode function can be set as follows:

(7) $$S_{1}\;=\;\dot{e_{s}}\;+\;\lambda_{1}e_{s},\;\lambda_{1}\;>\;0$$

The virtual control law $u_{s}$ of the position subsystem is designed as follows:

(8) $$u_{s}=\hat{m}{\bar{u}}_{s}-\hat{d_{s}}$$

${\bar{u}}_{s}$ is expressed as:

(9) $${\bar{u}}_{s}=ge_{3}+\ddot{\xi_{c}}-\lambda_{1}\;\dot{e_{s}}-c_{1}S_{1}$$

$c_{1}$ is the control coefficient, where $\;c_{1}>0$, $\hat{m}$ is an estimator of quality, $\hat{d_{s}}$ is an etimate of the disturbance force of the displacement part.

Calculating the first derivative of sliding mode function:

(10) $$\dot{S_{1}}\;=\;\ddot{e_{s}}\;+\;\lambda_{1}\dot{e_{s}}$$

(11) $$\dot{S_{1}}=\frac{\hat{m}{\bar{u}}_{s}-\hat{d_{s}}+d_{s}}{m}-ge_{3}-\ddot{\xi_{c}}+\lambda_{1}\dot{e_{s}}$$

Further sorting out:

(12) $$\dot{S_{1}}\;=\;\frac{\hat{m}{\bar{u}}_{s}-\;\hat{d_{s}}\;+\;d_{s}}{m}-{\bar{u}}_{s}-c_{1}S_{1}$$

Compared with other articles, in the position motion part, the main purpose of this paper is to reduce the influence of the disturbance torque on the motion trajectory, so the disturbance torque is designed as the adaptive law to better track the position trajectory. Simultaneously, in order to reflect the superiority of the control method, considering the change of the mass caused by the load of the aircraft, which will affect the lift dynamics and stability of the quadrotor aircraft, the mass adaptive law is also designed.

The adaptive law is defined as follows:

(13) $$\dot{\hat{d_{s}}}\;=\;\gamma_{1}S_{1}$$

(14) $$\dot{\hat{m}}=-\gamma_{2}S_{1}{}^{T}{\bar{u}}_{s}$$

$\gamma_{1}$ and $\;\gamma_{2}$ are control coefficients.

The disturbance force error of displacement part is defined as $e_{d_{s}}$=$\;d_{s}-\hat{d_{s}}$. The quality error is $e_{m}$ = *m*$-\hat{m}$.

Lyapunov function is defined as follows:

(15) $$V_{1}=\;\frac{1}{2}S_{1}{}^{T}S_{1}\;+\;\frac{1}{2m\gamma_{1}}e_{d_{s}}{}^{T}e_{d_{s}}\;+\;\frac{1}{2m\gamma_{2}}e_{m}{}^{2}$$

The derivation of Lyapunov function is obtained:

(16) $$\dot{V_{1}}=\;S_{1}{}^{T}\left(\frac{\hat{m}{\bar{u}}_{s}-\;\hat{d_{s}}\;+\;d_{s}}{m}-{\bar{u}}_{s}-c_{1}S_{1}\right)\;+\;\frac{1}{m\gamma_{1}}e_{d_{s}}{}^{T}{\dot{e}}_{d_{s}}+\frac{1}{m\gamma_{2}}e_{m}{\dot{e}}_{m}$$

The interference force and mass of the position part are defined as slow time-varying signals, so the mass error ${\dot{e}}_{m}$ is related to $\dot{\hat{m}}$ in adaptive control law ${\dot{e}}_{m}=-\dot{\hat{m}}$. By analogy, the position disturbance force error and $\dot{\hat{d_{s}}}$ in adaptive control law also exists as ${\dot{e}}_{d_{s}}$ = $-\dot{\hat{d_{s}}}$. The mass error of position interference force error is expressed by this relation, and then it is brought into the Lyapunov derivative function

(17) $$\dot{V_{1}}=-c_{1}S_{1}{}^{T}S_{1}-\frac{e_{d_{s}}{}^{T}}{m\gamma_{1}}(\dot{\hat{d_{s}}}-\gamma_{1}S_{1})-\frac{e_{m}}{m\gamma_{2}}(\dot{\hat{m}}+\gamma_{2}S_{1}{}^{T}{\bar{u}}_{s})$$

According to the above relations, $\dot{V_{1}}$ is approximately equal to $-c_{1}S_{1}{}^{T}S_{1}$. According to Lyapunov’s law [24], in order to ensure the system is in a stable state, it is necessary for $\dot{V_{1}}=-c_{1}S_{1}{}^{T}S_{1}\leq$ 0. The analysis shows that when the sliding mode function is not zero, the value of Lyapunov derivative function is always less than zero. According to the property of derivative function, the Lyapunov function decreases monotonically, and the sliding mode function, disturbance force error, and mass error decrease gradually. When the sliding mode function $S_{1}$ is equal to zero, the Liapunov derivative is equal to zero. At this time, the concept of LaSalle invariant set principle is introduced to analyze. According to the principle of LaSalle invariant set [25], it can be considered that the sliding mode function converges to zero gradually. When *t* →∞,$\;S_{1}\to 0$. Further, the sliding mode function $S_{1}$ is equal to zero, because the Lyapunov derivative function $\dot{V_{1}}\;$= 0, so the Lyapunov function is a constant, and $e_{d_{s}}$ and $e_{m}$ are invariant bounded quantities.

In this case, the sliding mode function will continue to cross at zero, causing the two error quantities to constantly change within a bounded interval, and there is no guarantee that the two error quantities will approach zero. In order to avoid the excessive amount of quality error, the input quantity $u_{1}$ needs to be continuously increased, so when the maximum and minimum values of the quality are known, the adaptive law of quality is corrected by a mapping adaptive algorithm [26].

(18) $$\dot{\hat{m}}=\mathrm{Pro}j_{\hat{m}}\left(-\gamma_{2}S_{1}{}^{T}{\bar{u}}_{s}\right)\;\mathrm{Pro}j_{\hat{m}}(.)=\{\begin{array}{c} 0,\;\mathrm{if}\;\hat{m}\geq m_{max}\mathrm{and}.>0\\ 0,\;\mathrm{if}\;\hat{m}\leq -m_{max}\mathrm{and}.<0,\;\\ \mathrm{other} \end{array}$$

When $\hat{m}$ is greater than the maximum value of the limit range and is in an increasing state, at this time, $\hat{m}$ remains unchanged, $\hat{m}$ is equal to 0. When $\hat{m}$ is lower than the minimum value of the limit range and is in a state of decreasing, at this time, $\hat{m}$ remains unchanged, and $\hat{m}$ is equal to 0. The mapping adaptive algorithm can not only ensure that $\hat{m}$ is equal to 0, but also ensures that $\dot{V_{1}}\leq$0.

In the case that the virtual control law $u_{s}$ can be calculated, the actual lifting power $u_{1}$ of the quadrotor and the sum $\eta_{s}$ of the three attitude angle signals of the attitude subsystem must be calculated. Therefore, the virtual control input quantity $u_{s}$ can be expressed as: ${\left[\begin{array}{ccc} u_{x} & u_{y} & u_{z} \end{array}\right]}^{T}$, and the intermediate quantity $\eta_{s}$ is expressed as the following form: ${\left[\begin{array}{ccc} \gamma_{s} & \theta_{s} & \psi_{s} \end{array}\right]}^{T}$.

Because $\;u_{s}\;$=$\;u_{1}R_{AB}e_{3}$, the matrix operation on $u_{s}$ is expressed as the following form, and the power input expression in the *x*, *y*, and *z* directions can be obtained by sorting.

(19) $$\left[\begin{array}{c} u_{x}\\ u_{y}\\ u_{z} \end{array}\right]=u_{1}R_{AB}\left[\begin{array}{c} 0\\ 0\\ 1 \end{array}\right]$$

(20) $$u_{x}=u_{1}\;\left(\cos\psi \cos\gamma \sin\theta +\sin\gamma \sin\psi \right)$$

(21) $$u_{y}=u_{1}\;\left(\sin\theta \sin\psi \cos\gamma -\sin\gamma \cos\psi \right)$$

(22) $$u_{z}=u_{1}\;\left(\cos\theta \cos\gamma \right)$$

The $u_{1}\;$= $\frac{u_{z}}{\cos\theta \cos\gamma }\;$ are substituted into expressions (21) and (22) and transformed into state space expression.

(23) $$u_{x}=u_{z}\left[\begin{array}{cc} \cos\psi & \sin\psi \end{array}\right]\left[\begin{array}{c} \tan\theta \\ \tan\gamma \sec\theta \end{array}\right]$$

(24) $$u_{y}=u_{z}\left[\begin{array}{cc} \cos\psi & -\sin\psi \end{array}\right]\left[\begin{array}{c} \tan\theta \\ \tan\gamma \sec\theta \end{array}\right]$$

Adding the intermediate amount $\eta_{s}$ leads to:

(25) $$\left[\begin{array}{c} u_{x}\\ u_{y} \end{array}\right]=u_{z}\left[\begin{array}{c} \begin{array}{cc} \cos\psi_{s} & \sin\psi_{s} \end{array}\\ \begin{array}{cc} \cos\psi_{s} & -\sin\psi_{s} \end{array} \end{array}\right]\left[\begin{array}{c} \tan\theta_{s}\\ \tan\gamma_{s}\sec\theta_{s} \end{array}\right]$$

(26) $$u_{x}\cos\psi_{s}+u_{y}\sin\psi_{s}=u_{z}\tan\theta_{s}$$

(27) $$u_{x}\sin\psi_{s}-u_{y}\cos\psi_{s}=u_{z}\frac{\tan\gamma_{s}}{\cos\theta_{s}}$$

The pitch angle and roll angle can be expressed as follows:

(28) $$\theta_{s}=\arctan\frac{u_{x}\cos\psi_{s}+u_{y}\sin\psi_{s}}{u_{z}}$$

(29) $$\gamma_{s}=\arctan\left(\cos\theta_{s}\frac{\;u_{x}\sin\psi_{s}-u_{y}\cos\psi_{s}}{u_{z}}\right)$$

The pitch angle signal $\theta_{s}$ and the roll angle signal $\gamma_{s}$ are used to track the reference position. They are generated by the virtual control input of the outer ring part and passed to the attitude subsystem of the inner ring part. The error generated by the outer ring part is processed by the inner ring part eliminate. The yaw angle $\psi_{s}$ is used as a given control signal and is generated by a signal generator. The input value of the signal generator can be set to track any yaw angle. The actual position controller input design is $u_{1}\;$=$\;u_{z}/\cos\theta \cos\gamma$, and $u_{1}$ is the actual control input.

### 3.3. Design of Attitude Tracking Controller

The attitude control part is the inner loop subsystem, which realizes attitude control through the inner loop control law, while tracking the pitch angle signal quantity $\theta_{s}$ and roll angle signal quantity $\gamma_{s}$ generated by the outer loop control. In the control of the attitude subsystem, the main tracking quantity is $\eta_{s}$. According to the expression of the posture part equation derived above, the controller of the attitude part is mainly designed for the control quantity input torque ***τ***. At the same time, the uncertainty in the quadrotor model and the external non-structural disturbance torque in the rotational motion are considered. Therefore, the expression of the attitude part of the quadrotor can be written as follows.

(30) $$J_{s}\ddot{\eta }=-C_{s}\dot{\eta }+\tau +d_{M}-J_{\Delta }\ddot{\eta }-C_{\Delta }\dot{\eta }$$

where ***J*** =$\;J_{s}\;$+ $J_{\Delta }$, ***C*** *=*$\;C_{s}$*+*$\;C_{\Delta }$. The interference force $d_{1}$ in the *x* direction can be expressed as an expression related to the interference torque. Further, consider that $d_{1}$ is a bounded quantity.

So, the dynamic model of the attitude subsystem can be expressed as:

(31) $$J_{s}\ddot{\eta }+C_{s}\dot{\eta }=\;\tau +\;d_{1}$$

The tracking error signal of the attitude subsystem is $e_{\eta }\;$=$\;\eta -\eta_{s}$, and the derivative is further expressed as $\dot{e_{r}}\;$=$\;\dot{\eta_{s}}-\lambda_{2}e_{\eta }$, $\eta_{s}$=${\left[\begin{array}{ccc} \gamma_{s} & \theta_{s} & \psi_{s} \end{array}\right]}^{T}$.

Similar to the design idea of the position controller, first define the sliding mode function. The sliding mode function $S_{2}$ is set to the following form:

(32) $$S_{2}=\dot{\eta }-\dot{e_{r}}=\dot{e_{\eta }}+\lambda_{2}e_{\eta }$$

$\lambda_{2}$ is the control parameter.$\;\lambda_{2}\;$ > 0.

According to the defined sliding mode function relationship, the attitude error subsystem can be transformed into the following form:

(33) $$J_{s}\dot{S_{2}}=\;\tau +\;d_{1}-C_{s}\dot{\eta }-J_{s}\ddot{e_{r}}$$

Therefore, the control quantity input torque can be expressed as the following form:

(34) $$\tau \;=\;J_{s}\ddot{e_{r}}\;+\;C_{s}\dot{\eta }-c_{2}S_{2}-\varepsilon sgn\left(S_{2}\right)$$

In Formula (34): *ε* and $c_{2}$ are control parameters, ε >$\;d_{\gamma }$,$\;c_{2}\;$ > 0. sgn is a symbolic function.

It can be seen from the expression of the exponential reaching law that when the exponential approaching speed is gradually reduced from a larger value to zero, the reaching time becomes shorter and the speed of the target point approaching the switching plane is very small. Because it is a process of gradual approximation, it is difficult to ensure that the moving point reaches the switching plane in a certain time, so an isokinetic approach term $-\varepsilon sgn\left(S_{2}\right)$ is added to the above-mentioned basis [18,19].

The nominal values of the defined model are:

(35) $$J_{S}=\left[\begin{array}{ccc} I_{x} & 0 & 0\\ 0 & I_{y}+I_{Z} & 0\\ 0 & 0 & I_{x}+I_{y}+I_{Z} \end{array}\right]\;C_{s}\;=\;0.9C$$

Furthermore, the Lyapunov function is constructed as follows:

(36) $$V_{2}\;=\;\frac{\;1}{\;2}S_{2}{}^{T}J_{S}S_{2}$$

The derivative function of $V_{2}$ can be obtained as follows:

(37) $$\dot{V_{2}}=\;S_{2}{}^{T}J_{S}\dot{S_{2}}\;=\;S_{2}{}^{T}(\tau \;+\;d_{1}-C_{s}\dot{\eta }-J_{s}\ddot{e_{r}})$$

After the control quantity τ is brought in, the following results can be obtained:

(38) $$\dot{V_{2}}=\;-2c_{2}J_{S}{}^{-1}V_{2}$$

It can be seen that Lyapunov derivative function $\dot{V_{2}}$ is a negative number, so the characteristic of Lyapunov function is monotonic decreasing. According to Lyapunov stability law, the attitude subsystem can be considered as stable, and the error is convergent.

For the whole system, the Lyapunov function is defined as:

(39) $$V=\;V_{1}+\;V_{2}$$

Put $V_{1}$ and $V_{2}$ in it.

(40) $$\dot{V}\text{≤}-c_{1}S_{1}{}^{T}S_{1}-c_{2}{}^{’}V_{2}\leq \;0$$

According to the Lyapunov stability law, the whole system can be considered to be in a state of asymptotic stability.

The position controller obtains the pitch signal $\theta_{s}$ and roll signal $\gamma_{s}$. From the expression of input torque in formula (34), it can be seen that the first derivative and the second derivative of the two signals need to be calculated respectively. In this case, we can use the form of third-order differentiator to realize the control of $\theta_{s}$ and $\gamma_{s}$ first derivative and second derivative.

## 4. Simulation Research

### Simulation

First of all, given the theoretical inspection trajectory parameter $\xi_{c}$ of the inspection drone, the theoretical trajectory is: *x* = cos(1/2*t*); *y* = sin(1/2*t*); *z* = *t*. Due to the under-driving characteristics of the inspection drone, the pitch angle and roll angle are transmitted to the inner loop control system as intermediate command signals, so only the yaw angle needs to be defined at this time. Set the constant of yaw angle to $\psi_{s}\;$= π/6. The distance from the end of the rotor to the center of gravity of the aircraft is 0.5 m. The parameters in the inertia matrix ***I*** are set as follows:$\;I_{x}\;$= 0.121; $I_{y}\;$= 0.121; $I_{z}$ = 0.151.

Interference force in displacement motion $d_{s}$ is:

$$\left[\begin{array}{c} \sin\left(\pi t\right)\\ \cos\left(\pi t\right)\\ \cos\left(\pi t\right) \end{array}\right]$$

Disturbing torque in rotating motion $d_{M}$ is taken as:

$$\left[\begin{array}{c} \sin\left(0.5\pi t\right)+0.5\\ \cos\left(0.5\pi t\right)+0.5\\ \sin\left(0.5\pi t\right)+0.6 \end{array}\right]$$

Set the simulation time to 30 s and change once in 10 s. In 0–10 s, *m* is 1 kg; in 10–20 s, *m* is 3 kg; in 20–30 s, *m* is 5 kg. In the position tracking controller, $c_{1}$ = 6, $\lambda_{1}$= diag [2, 2, 2], $\gamma_{1}=0.80,\gamma_{2}=0.30$. In the attitude controller, $c_{2}\;$= 8, ε = 0.30, $\lambda_{2}\;$= diag [60, 60, 60]. In the switching control process, the saturation function is used instead of the switching function, and the boundary layer thickness is taken as 0.30.

The simulation results are shown in the figure.

**Figure 5:**
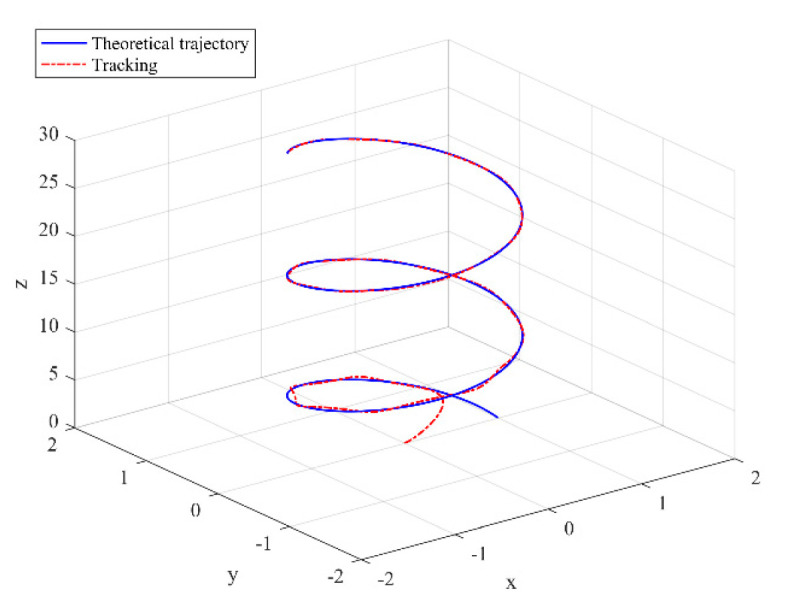
Trajectory tracking effect chart.

**Figure 6:**
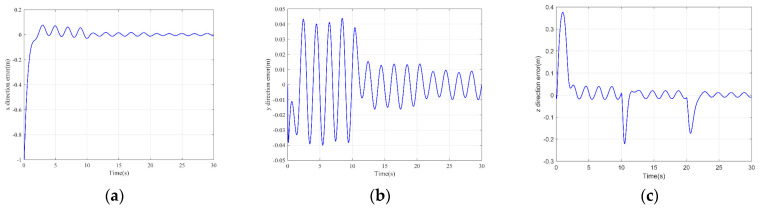
Position tracking error. (**a**) *x* direction position error; (**b**) *y* direction position error; (**c**) *z* direction position error.

**Figure 7:**
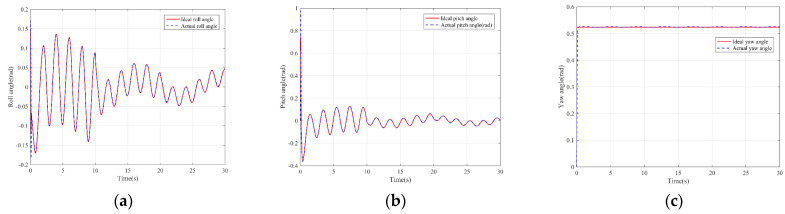
Posture tracking effect diagram. (**a**) Track of roll angle *γ* (**b**) Pitch angle *θ* track tracking (**c**) Yaw angle *ψ* trajectory tracking.

**Figure 8:**
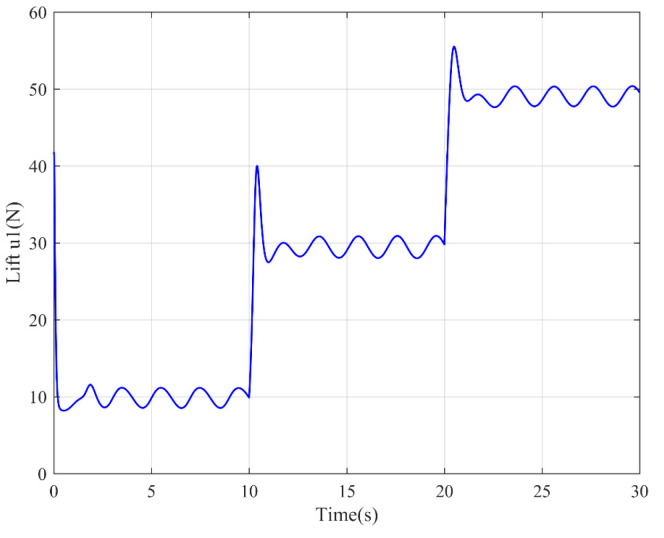
Lifting power of drone.

**Figure 9:**
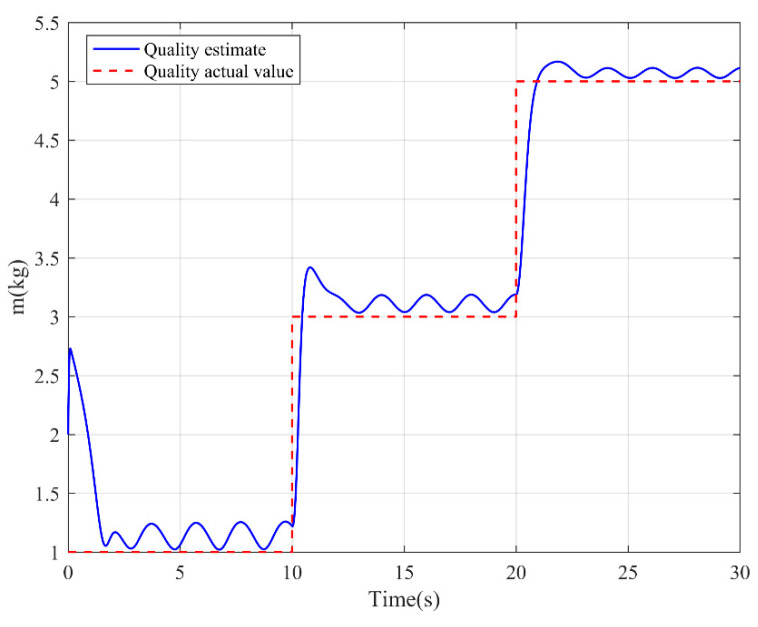
Mass adaptive estimation.

**Figure 10:**
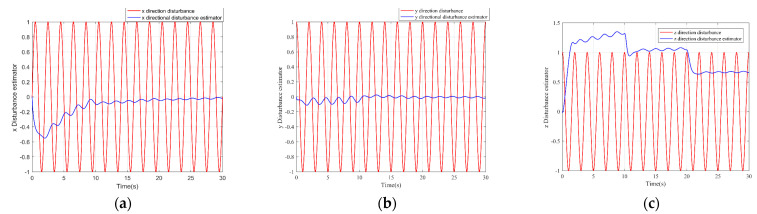
Adaptive estimation of position direction interference force. (**a**) *x* direction disturbance adaptive effect diagram; (**b**) *y* direction disturbance adaptive effect diagram; (**c**) *z* direction disturbance adaptive effect diagram.

It can be seen from Figure 5 that the inspection drone can track the inspection trajectory well. The blue is the theoretically designed inspection trajectory, and the red is the actual tracking trajectory under the disturbance of the disturbance. It can be seen from the figure The two trajectories are close to the same, which shows the effectiveness of the control algorithm proposed in this paper. Figure 6a–c present the tracking errors in the *x*, *y*, and *z* directions of the inspection drone, respectively. It can be seen that after the quality is changed, the error changes in different time periods are different, and the error is very small and within a reasonable range. Figure 7a–c are the trajectory tracking curves of the roll angle, pitch angle, and yaw angle of the inspection drone. It can be seen that the actual roll angle, pitch angle, and the yaw angle and the ideal roll angle, pitch angle, and yaw angle curve basically tend to be the same. Figure 8 shows the change in the lifting power of the inspection drone, i.e., the input quantity $u_{1}$. From the figure, it can be seen that the lift changes with the change of mass, and the value is close to the gravity of the inspection drone. Figure 9 shows the quality self-adaptive estimation result. The quality estimation value and the actual quality value curve are roughly the same, and the error is within the allowable range. Figure 10 shows a comparison diagram of the disturbance force design value and estimated value of the position movement part. It can be clearly seen from the figure that the disturbance force error in the *x*-direction and *y*-direction basically approaches zero, achieving the expected goal. Compared with the designed interference force, the *z* direction is significantly reduced, and the disturbance is suppressed. The effectiveness and robustness of this control algorithm can also be seen from the before and after changes of the interference force. In practical experiments, the gust of wind can be regarded as a disturbance, and the disturbance can be well suppressed by the adaptive law.

Through the above simulation analysis and research, it can be seen that the controller designed by the sliding mode adaptive robust control method can accurately and stably track the given patrol, taking into account the dynamic characteristics of the patrol drone, external disturbance, and system uncertainty. Check the trajectory, obtain good control performance, and verify the effectiveness of the control algorithm.

## 5. Experimental Design

### 5.1. Sliding Mode PID Control Group Experiment

**Figure 11:**
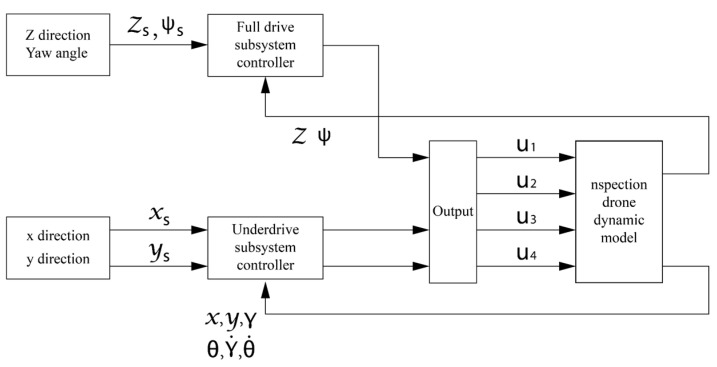
Structure chart of sliding mode PID control.

The first group of control experiments uses the sliding mode PID control algorithm to design the inspection drone controller. The control structure diagram is shown in Figure 11.

The system is divided into two parts, one is a full drive subsystem, the other is the under drive subsystem. PID control is used in the full drive subsystem and sliding mode control is used in the underactuated subsystem. When other conditions remain unchanged, the mass is 1 kg for the control experiment. The simulation results are shown in the figure.

**Figure 12:**
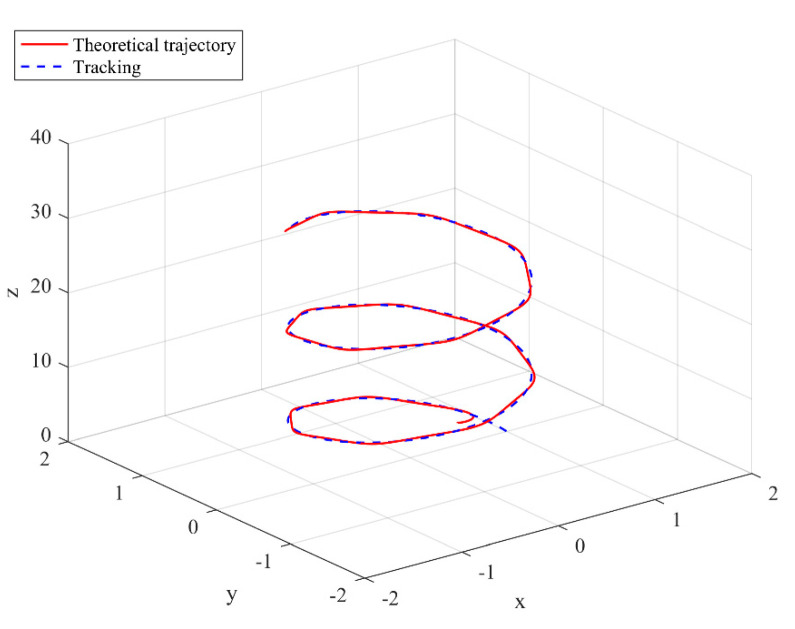
Trajectory tracking effect chart.

**Figure 13:**
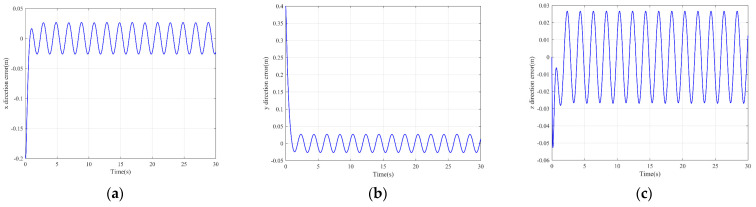
Position tracking error. (**a**) *x* direction position error; (**b**) *y* direction position error; (**c**) *z* direction position error.

**Figure 14:**
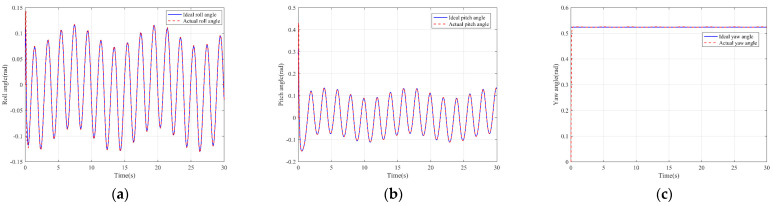
Posture tracking effect diagram. (**a**) Track of roll angle *γ*; (**b**) Pitch angle *θ* track tracking; (**c**) Yaw angle *ψ* trajectory tracking.

From the simulation results of the sliding mode PID control algorithm, it can be seen from Figure 12 that the overall trajectory tracking has a good effect. The specific position tracking amount is shown in Figure 13. It is not difficult to see from the figure that the error in the *x* direction is between −0.03 and 0.03; the error in the *y* direction is between −0.02 and 0.02; the error in the *z* direction is between −0.026 and 0.026. Although the error is oscillating, it is higher in terms of accuracy than the control algorithm proposed in this paper. Figure 14 presents the attitude tracking curves. It can be seen that the overall trajectory tracking accuracy has a good effect, which is equivalent to the control effect in this article.

### 5.2. Backstepping Control Group Experiment

**Figure 15:**
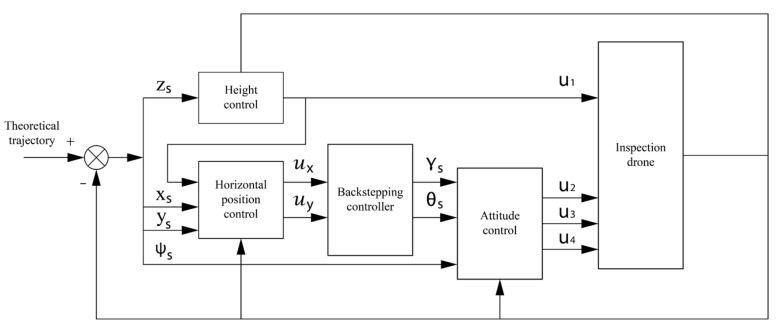
Structure chart of backstepping control.

The second group of control group experiments adopts backstepping control to design the controller. The control structure diagram is shown in Figure 15. The basic principle of backstepping is to decompose a complex nonlinear system into subsystems that do not exceed the order of the system, and then design partial Lyapunov functions and intermediate virtual control variables for each subsystem, and “backward” to the entire system. They are integrated to complete the design of the entire control law. Therefore, the overall system of the inspection UAV can also be divided into two parts, namely the position subsystem and the attitude subsystem. Firstly, the theoretical inspection trajectory of the inspection drone is given. At the same time, the height control uses the error amount between the actual height and the theoretical height as input, and the lift $u_{1}$ in the *z* direction is obtained through the calculation of the controller. Similarly, in the translation part, the theoretical values and actual errors in the *x* and *y* directions are used as input, and the lift $u_{1}$ in the *z* direction is brought into the translation controller to calculate the lift $u_{x}$ and $u_{y}$ in the two directions. After $u_{x}$ and $u_{y}$ undergo backstepping control operations, the theoretical roll angle and theoretical pitch angle of the system are obtained. The second is the design of the attitude subsystem, which is similar to the design of the position controller. The theoretical value of the three angles of the attitude part and the actual value measured by the attitude sensor are calculated to obtain the error, and the error is used as the input. It should be noted that the yaw angle is still set at this time. The three angle errors are input into the attitude controller for calculation, and the input torques $u_{2}$, $u_{3}$, and $u_{4}$ of the three angles are obtained. Finally, the input lift and torque obtained by the two subsystems are used to control the work of the inspection drone. The simulation results are shown in the figure.

**Figure 16:**
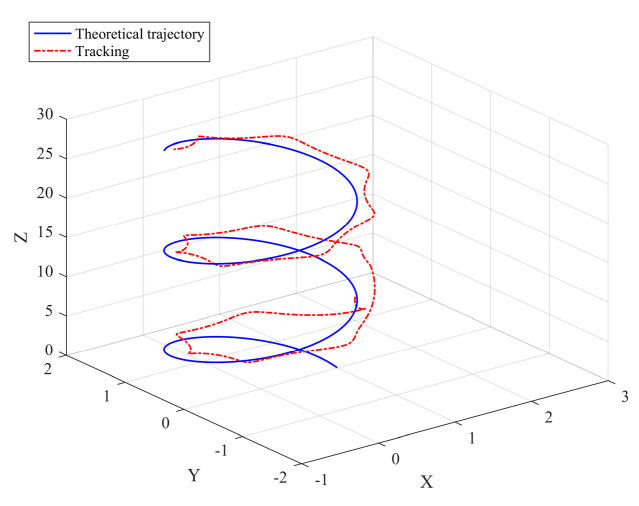
Trajectory tracking effect chart.

**Figure 17:**
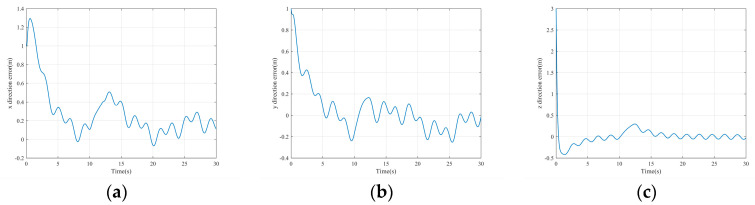
Position tracking error. (**a**) *x* direction position error; (**b**) *y* direction position error; (**c**) *z* direction position error.

**Figure 18:**
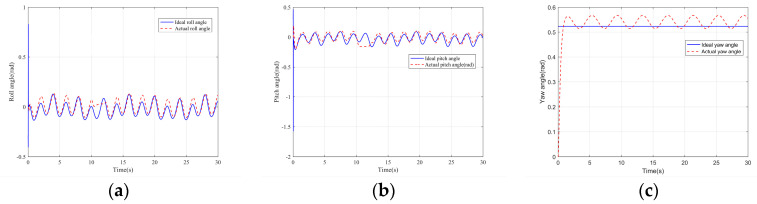
Posture tracking effect diagram. (**a**) Track of roll angle *γ*; (**b**) Pitch angle *θ* track tracking; (**c**) Yaw angle *ψ* trajectory tracking.

It can be seen from Figure 16 that due to the addition of disturbance, the overall trajectory tracking effect is not ideal, and the disturbance is relatively serious. Looking at the error change curves in the three directions, the overall error curve is in an oscillating state, and the oscillating range is relatively large, which shows that the control algorithm is greatly affected in terms of accuracy. It can be seen from Figure 17 and Figure 18 that the tracking effect of the three angles is affected by the disturbance and the angle tracking cannot be performed well.

## 6. Experimental Study

**Figure 19:**
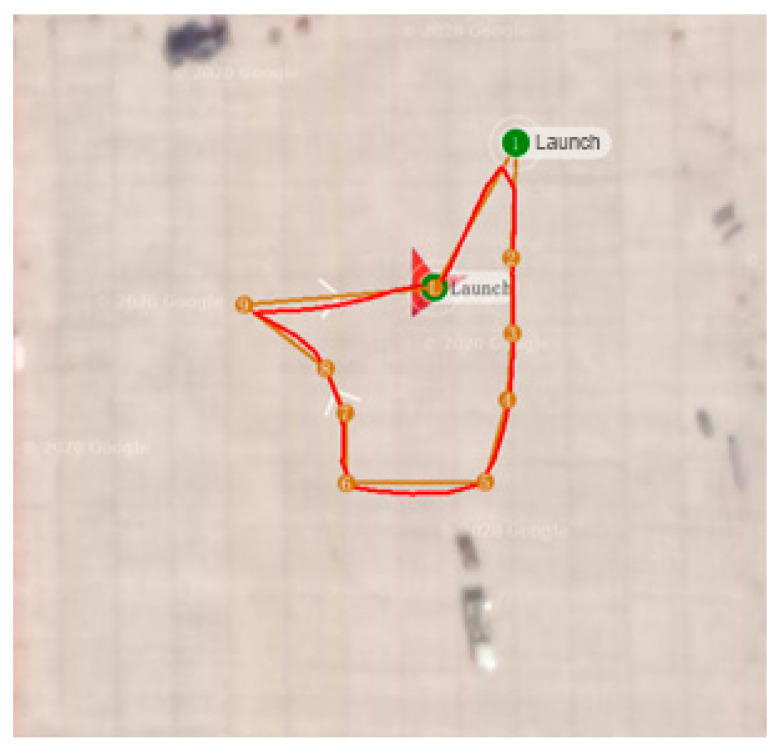
Inspection track design.

In order to better verify the effectiveness of the proposed control algorithm, this paper adds an actual experimental platform for verification. The hardware system part of the experimental platform is the PIX series flight controller with the model of the main control chip STM32F427. The MPU6050 sensor is selected as the attitude acquisition component. The GPS module with the model number M8N and the MS5611-01BA air pressure sensor are selected for the measurement of position parameters. The experiment mainly uses wind as interference to measure the flight status and trajectory tracking of the UAV to verify the superiority of the control strategy proposed in this paper. The patrol track of the experimental design is shown in Figure 19.

**Figure 20:**
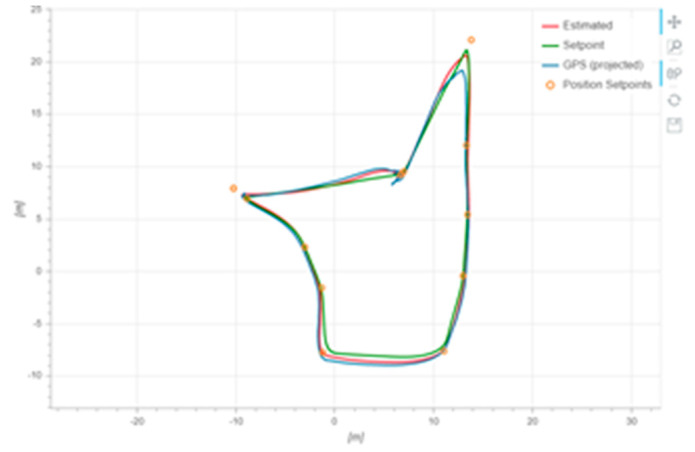
Theoretical and practical trajectories.

**Figure 21:**
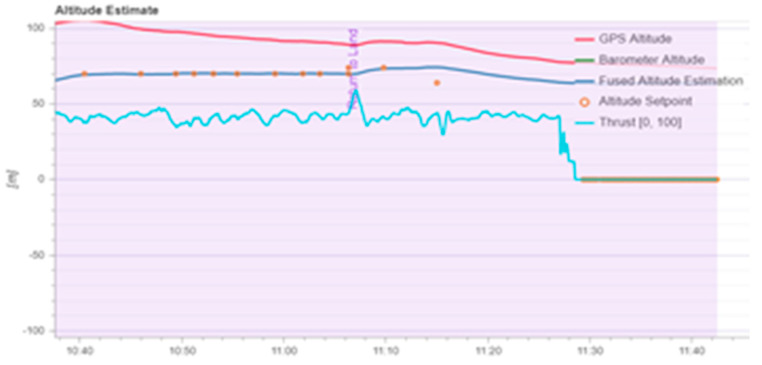
Altitude data from aircraft and GPS.

**Figure 22:**
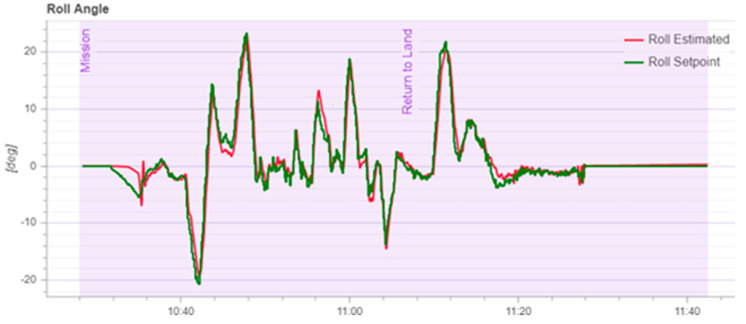
Schematic diagram of tracking results for cross-roll angle.

**Figure 23:**
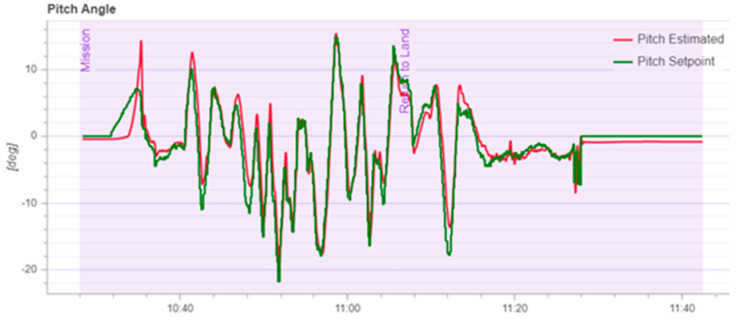
Schematic diagram of tracking results for pitch angle.

**Figure 24:**
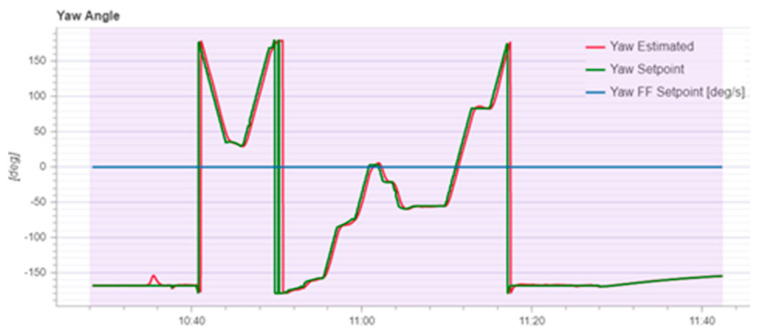
Schematic diagram of tracking results for pitch angle.

**Figure 25:**
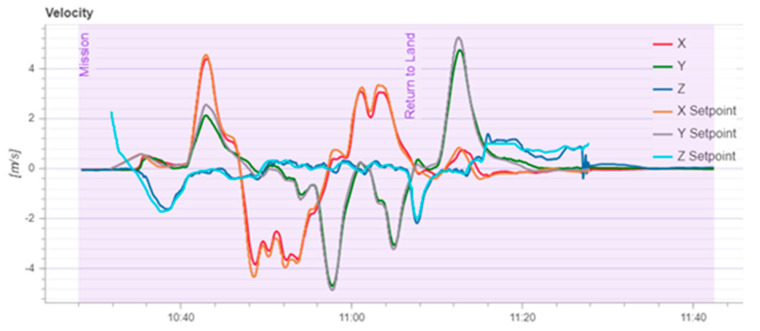
Location track tracking effect.

**Figure 26:**
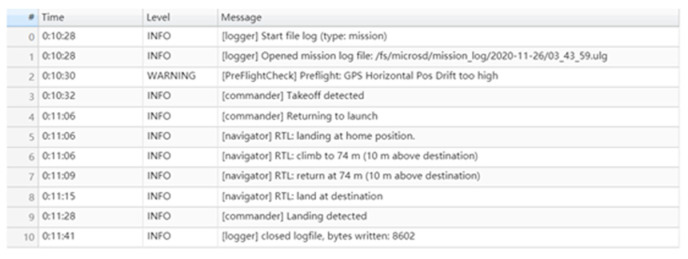
Speed variation of inspection drones.

According to Figure 20, it can be seen that after designing the predetermined trajectory, the inspection drone can roughly track the predetermined trajectory, and although there is an error in the tracking result, considering the influence of wind gusts and disturbances during the experiment, the tracking result is also within the controllable range. The experimental data from Figure 21, Figure 22, Figure 23, Figure 24, Figure 25 and Figure 26 can clearly show the tracking results of each index, and the actual value curve and the theoretical value basically tend to be the same, which shows the effectiveness of the control algorithm, and the error range of each index is also within the plan.

In order to better compare the three control algorithms during the experiments, different algorithms are used for the control experiments. The parameters of the experimental equipment are kept constant and only the control algorithms are changed. The experimental results of the three control algorithms are shown in Table 1.

Combining the experimental data in Table 1 and Table 2, it can be seen that the sliding mode adaptive robust control algorithm is faster in terms of stabilization time than the sliding mode PID control algorithm and the backstepping control algorithm. Although the two algorithms used as the control group can finally track the motion trajectory of the quadrotor aircraft, in terms of tracking error, the tracking error range of the two algorithms is larger than that of the sliding mode adaptive robust control algorithm. Although the sliding mode PID algorithm can achieve good results in respect of stability time and tracking accuracy, it is very cumbersome to adjust the PID parameters, and the overshoot phenomenon is serious when the parameters are not selected properly. It can be concluded that the sliding mode adaptive robust control algorithm has good stability, higher precision, and obvious advantages by comparing the experimental results of the other two control algorithms under the condition of considering the disturbance.

## 7. Discussion

Although the simulation results can basically achieve the expected goal, the error in the *z*-direction is significantly different compared to the error variation range in the *x* and *y* directions. Moreover, the final obtained disturbance moment in *z*-direction is also significantly different compared to the other two directions, which shows that the accuracy and the suppression of disturbance in *z*-direction still need to be improved. Further analysis of the causes of this situation may be as follows.

1. The inspection drone is an underdriven system where the control volume is less than the output volume.
2. Jitter vibration will be generated near the transition to the slide surface during the control process.
3. The actual altitude always has a constant small deviation from the ideal altitude after the trajectory tracking process is stabilized.
4. The inner loop of attitude control and the outer loop of position control cannot be coordinated immediately during the continuous steering process.

## 8. Conclusions

The results show that the sliding mode adaptive robust control strategy used in this paper can support the inspection UAV to track the inspection trajectory well. In the presence of interference, the stability and accuracy of the inspection UAV are guaranteed. Simulation results and experimental results prove the effectiveness of the control method. The simulation experiments of the control group prove the advantages of the control strategy in this study. At the same time, in the process of comparison with the sliding mode PID control and the backstepping control, it can be clearly seen that the sliding mode PID can achieve reasonable results in terms of control accuracy, and the backstepping control, although designed with system stability in mind, struggles to achieve the expected results in terms of control accuracy. In the control process of the actual experiment, from the data measured by the sensor and the theoretical data curve calculated on the ground station, it can be seen that the control algorithm used can reach the actual demand, and the inspection drone can follow the control point well for inspection. In the future, we will continue to combine the algorithm from the anti-interference and the inspection drone in order to better apply to the actual project.

## Figures and Tables

**Table 1:** Control algorithm error tracking result analysis table.

Control Method	*x*-Direction Tracking Error Range	*y*-Direction Tracking Error Range	*z*-Direction Tracking Error Range
Sliding Mode Adaptive Robustness	−0.21–0.16	−0.31–0.58	−0.63–1.21
Sliding Mode PID	−0.23–0.36	−0.86–0.79	−0.59–1.32
backstepping control	−0.56–1.21	−1.52–1.63	−1.63–1.82

**Table 2:** Attitude trajectory tracking error.

Control Method	Roll Angle	Pitch Angle	Yaw Angle
Sliding Mode Adaptive Robustness	−0.5–0.75	−0.04–0.58	−0.21–0.36
Sliding Mode PID	−0.86–0.95	−0.5–0.36	−0.31–0.48
backstepping control	−0.98–1.025	−0.93–0.86	−1.26–1.10
